# From threat to strength: how AI usage is paradoxically associated with resilience among accounting and finance employees with perceived work meaningfulness as a mediating mechanism

**DOI:** 10.3389/fpsyg.2026.1820550

**Published:** 2026-07-13

**Authors:** Yuanyuan Ji, Huaming Wu

**Affiliations:** 1 School of Smart Finance and Business, Hefei College of Finance and Economics, Hefei, China; 2 School of Business Administration, Anhui University of Finance and Economics, Bengbu, China

**Keywords:** artificial intelligence usage, employee resilience, job complexity, job demands-resources theory, perceived work meaningfulness

## Abstract

**Introduction:**

Artificial intelligence is increasingly reshaping accounting and finance work, yet its psychological implications for employees remain insufficiently understood. While existing studies have mainly emphasized the productivity and efficiency outcomes of AI, less is known about how AI usage relates to employee resilience in accounting and finance roles. To address this gap, this study examines the relationship between AI usage and employee resilience and explores the psychological mechanism and boundary condition underlying this relationship.

**Methods:**

Based on the Job Demands-Resources (JD-R) theory, this study develops a moderated mediation model in which perceived work meaningfulness acts as the mediator and job complexity serves as the moderator. Data were obtained from employees in accounting and finance-related positions through a three-wave time-lagged survey. After matching responses across the three waves and screening invalid cases, 332 valid questionnaires were used for analysis.

**Results:**

The results showed that AI usage positively predicted employee resilience, and perceived work meaningfulness mediated this relationship. Job complexity further moderated the relationship between AI usage and perceived work meaningfulness, such that the positive relationship was stronger under conditions of low job complexity.

**Discussion:**

This study extends the JD-R framework to AI-enabled work contexts by conceptualizing AI usage as a work-related resource associated with employee resilience. The findings identify perceived work meaningfulness as a key psychological mechanism and job complexity as an important boundary condition, providing theoretical and practical implications for AI implementation in accounting and finance settings.

## 1 Introduction

Artificial intelligence has become increasingly embedded in organizational work processes. To improve efficiency and support innovation, many organizations have introduced AI into daily operations (Chui et al., 2016; Kaplan and Haenlein, 2018). This change is particularly noticeable in the fields of accounting and finance. AI-driven tools are widely applied in core accounting and finance tasks, including automated financial reporting, fraud detection, algorithmic risk assessment, automatic account reconciliation, audit tracking, anomaly identification, and intelligent decision support. Such tools assist employees in completing both routine operations and cognitively challenging professional work, thus triggering fundamental thinking: does this technological transformation undermine or support employees’ resilience?

As the core ability of individuals to maintain psychological stability and achieve adaptive recovery in adversity or change, employee resilience (ER) has been regarded as an indispensable psychological resource in the process of organizational transformation (Luthans et al., 2006). Existing studies are gradually focusing on the role of leadership and organizational culture in shaping resilience (Luthans and Youssef, 2007), but the potential role of AI as an antecedent associated with ER has not received enough attention. As AI gradually penetrates the daily workflow of accounting and financial practitioners, clarifying whether and how it can cultivate employees’ psychological resources has shown a strong urgency at the two levels of theoretical construction and practical guidance. Although the body of literature investigating the associations between AI usage, perceived work meaningfulness, and ER has been expanding, research on the moderating role of job complexity in this context remains insufficient; thus, this study is designed to address this gap.

A key psychological pathway linking AI usage to employee resilience may lie in perceived work meaningfulness (PWM), which refers to employees’ subjective perceptions of the meaning, value, and purpose of their work (Steger et al., 2012). The application of artificial intelligence not only changes the way of working (Meneses and Varajão, 2022), but also reshapes the model of employee experience and evaluation of work (Attaran et al., 2020). Research shows that when employees interact with artificial intelligence systems, the convenience and support provided by AI may be linked to higher PWM (Zirar et al., 2023). Therefore, when AI undertakes repetitive low-value tasks such as routine data entry or rule-based compliance checks, which are common in financial positions, employees can free up their energy to engage in activities with more cognitive challenges and judgment requirements, thus enhancing the sense of work significance. Wrzesniewski and Dutton (2001) suggested that PWM is closely related to employees’ intrinsic motivation and work performance. In addition, when employees recognize the value of new technologies for their positions, they may develop a stronger sense of purpose and value in their work (Rosso et al., 2010). Conversely, when AI threatens role identity or makes professional skills seem obsolete, it may weaken the basis on which employees derive meaning from work (Huang and Rust, 2018). However, in different work scenarios, this effect may not be the same. Job Complexity (JC) refers to the degree to which job tasks require advanced cognitive skills, independent decision-making capabilities, and the ability to handle non-routine work content (Morgeson and Humphrey, 2006), and it may serve as a critical boundary condition. The highly complex working environment requires employees to invest more cognitive and emotional resources, and there will be qualitative differences when using AI tools (Bai et al., 2021). Complex tasks will also push employees to consider the meaning and value of work, thus affecting job satisfaction and psychological resilience (Sonnentag and Fritz, 2007). In low-complexity positions, AI’s automation of daily tasks may be directly transformed into PWM promotion; while in high-complexity positions, because meaningful work is embedded in it, the incremental contribution of AI may be relatively weakened - and may even be regarded as erosion of professional identity (Huang and Rust, 2018).

In order to clarify the relationship, we drew on the Job Demands-Resources (JD-R) framework (Bakker and Demerouti, 2007; Demerouti et al., 2001). The framework points out that artificial intelligence has dual characteristics. It is not only a resource to reduce the cognitive load and improve the quality of decision-making (Davenport and Ronanki, 2018), but also a challenge to adapt to pressure and work security anxiety, which is particularly prominent in the digital financial environment (Acemoglu and Restrepo, 2020). This study makes three contributions to the existing literature: First, by developing and empirically testing a moderated mediation model, this study links AI usage with ER and extends the JD-R framework to AI-enabled work contexts in accounting and finance. Secondly, by identifying PWM as a mediating mechanism, this study shifts attention from the efficiency-related outcomes of AI to employees’ internal work experiences. Third, by introducing the complexity of work as a boundary condition, this study provides a more nuanced explanation of when AI usage may be associated with psychological resilience, thereby deepening our understanding of the contingent relationship between technology use and wellbeing among accounting and finance professionals.

**Figure 1:**
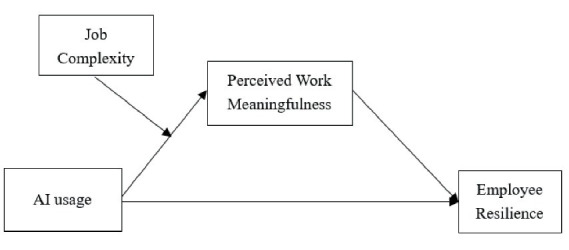
Research model.

Drawing from the arguments above, the following model is put forward in this study (Figure 1):

## 2 Theoretical background and hypotheses

### 2.1 Job demands-resources theory

JD-R theory suggests that job characteristics can be categorized into job demands and work resources. Job demands involve continuous work efforts at the physical, cognitive, and emotional levels, and are accompanied by physiological and psychological costs. In contrast, work resources help employees achieve work goals, reduce the costs associated with job demands, and foster personal growth and development. Through the motivational process, work resources can elicit positive psychological states (e.g., dedication and meaningfulness), thereby producing adaptive outcomes, including psychological resilience. Within this framework, artificial intelligence (AI) also exhibits dual characteristics. On the one hand, AI serves as a critical work resource that can optimize workflows and improve decision-making quality. On the other hand, AI may function as a potential job demand, bringing learning costs and adaptation pressure. Although AI possesses both attributes, the present study primarily draws on the motivational pathway of JD-R theory to examine AI’s positive functional value. Specifically, this study uses the JD-R motivational path to explain how AI usage may relate to ER by reshaping work meaningfulness. Moreover, JC is introduced as an important boundary factor to contextualize and moderate the relationship between AI usage and work meaningfulness.

Specifically, in this study, AI usage refers to the extent to which accounting and finance employees apply AI-enabled tools in their core daily work, including automated financial reporting, fraud detection, algorithmic risk assessment, automatic account reconciliation, audit tracking, anomaly identification, and intelligent decision support. These AI applications support employees in completing both routine and highly cognitive professional tasks to improve work efficiency and accuracy, while excluding general-purpose or non-job-related AI tools.

### 2.2 AI usage and employee resilience

Building on the JD-R theory, work resources refer to those environmental characteristics that help achieve goals, buffer the negative impact of demand and support personal growth (Demerouti et al., 2001). AI has fundamentally reconstructed the balance between resources and demand in the workplace by automating routine processes and providing intelligent decision-making support. Studies have shown that AI, as an efficient working resource, can effectively reduce the cognitive load in demanding working environments (Hassani et al., 2020). For accounting and financial personnel, an AI system that can automate account reconciliation, generate audit tracking or mark abnormal situations is an efficient working resource, which directly alleviates the high-intensity task burden that may have exhausted psychological resources.

Employee resilience refers to the ability to adapt, restore and maintain positive functions in adversity, and is particularly sensitive to such changes in work resources (Luthans et al., 2006). When AI reduces the burden of repetitive low-value tasks, employees can more effectively invest cognitive and emotional resources in high-level problem solving, thereby supporting employees’ psychological adaptability. In addition, real-time information and decision-making support provided by AI enable employees to cope with workplace challenges with greater confidence and agility (Jin et al., 2024), helping them manage stress more effectively and maintain positive performance under difficult conditions (Hetzel-Riggin et al., 2020). Thus, we propose:

*H1:* AI usage is positively associated with ER.

### 2.3 AI usage and perceived work meaningfulness

PWM, that is, employees’ subjective feelings about the meaning, purpose and value of their work (Steger et al., 2012), is the core driving force of intrinsic motivation, work performance and organizational effectiveness (Wrzesniewski and Dutton, 2001; Slemp and Vella-Brodrick, 2014). The application of artificial intelligence can improve PWM through multiple complementary channels. First of all, by automating tedious and repetitive tasks, AI enables employees to focus on complex and creative work that matches their core competencies (Jarrahi, 2018). Secondly, AI decision-making support enhances the sense of self-efficacy and promotes more active work evaluation (Cao et al., 2023). Third, the application of artificial intelligence may change employees’ perception of work and shift attention from program execution to high-level judgment and creativity, thus affecting PWM at a deeper cognitive level (Kubiak, 2020). These mechanisms together show that the effective use of artificial intelligence can enrich the quality of work in a way that deepens the sense of meaning of employees (Bai et al., 2024). With this in mind, we propose:

*H2:* AI usage is positively correlated with employees’ PWM.

### 2.4 The mediating role of perceived work meaningfulness

The previous discussion suggests that AI usage may be associated with ER partly through PWM. When work resources enrich the inner quality of work, they will generate a positive psychological state, thus improving employees’ ability to cope with adversity (Bakker and Demerouti, 2007). When the introduction of AI can help accountants focus on analytical judgment, customer consultation or strategic planning, and get rid of the mechanical way of handling work, they can better maintain motivation, persevere in setbacks, and maintain psychological stability under pressure (Steger et al., 2012). As employees participate in more value-added activities with the support of AI, their sense of achievement will deepen, and their resilience in the face of workplace challenges will also be enhanced (Sun et al., 2026; Chung et al., 2025). In other words, AI usage may not be directly associated with resilience, but may first change employees’ experience of work by making it more targeted, more attractive, and more conducive to psychological growth. Therefore, we propose:

*H3*: PWM serves as a mediator in the relationship between AI usage and ER.

### 2.5 Job complexity as a moderator

JC refers to the extent to which work tasks require diversified knowledge integration, independent decision-making, and the handling of non-routine content (Morgeson and Humphrey, 2006). High-complexity work environments require continuous investment of cognitive and emotional resources, leading employees to form distinctly different AI experiences compared with those in low-complexity jobs (Bai et al., 2021).

In low-complexity work settings, tasks are highly repetitive with low cognitive demands. AI effectively automates trivial and repetitive workflows, reduces unnecessary work consumption, and allows employees to concentrate on valuable and intrinsically rewarding job responsibilities, thereby significantly improving their PWM (Zhang et al., 2025). In contrast, in highly complex occupations such as senior auditing, financial analysis, and strategic consulting, employees engage in highly cognitive and autonomous professional work. Such work itself fosters a strong sense of mission and professional competence, with solid intrinsic work meaning already established (Morgeson and Humphrey, 2006). Thus, the incremental contribution of AI to PWM is therefore comparatively attenuated. Furthermore, in highly specialized and complex roles, AI intervention in professional judgment is more likely to be perceived as an erosion of professional identity and intrinsic work value rather than functional empowerment (Huang and Rust, 2018). Accordingly, we propose:

*H4:* JC moderates the relationship between AI usage and PWM, such that the positive relationship is stronger when JC is low rather than high.

Integrating the preceding hypotheses, we propose a moderated mediation model in which the indirect association between AI usage and ER depends on JC, with PWM serving as the mediator. H1 and H2 suggest positive links between AI usage, ER, and PWM; H3 identifies PWM as the mediating psychological mechanism; and H4 specifies JC as the boundary condition regulating the AI usage–PWM relationship.

When JC is low, AI usage is expected to show a stronger positive relationship with PWM, which may be reflected in a stronger indirect relationship with ER. When JC is high, the same indirect association is expected to be weaker, as the additional psychological value associated with AI usage may be more limited in highly complex jobs. Accordingly, we propose:

*H5*: JC moderates the indirect association between AI usage and ER, with PWM serving as the mediator, such that the indirect association is stronger when JC is low rather than high.

## 3 Methods

### 3.1 Participants and procedure

The target participants of this study are employees who hold accounting and finance-related positions across various industries. Screening items were set at the beginning of the questionnaire to ensure that only participants engaged in formal accounting and financial work could complete the survey, and responses from non-financial positions and irrelevant occupations were eliminated to unify the occupational attributes of the sample and match the research setting of finance and accounting employees. Questionnaire data were collected electronically through the Credamo platform. Before filling out the questionnaire, participants were told that their answers would remain anonymous and that nothing they shared would be traceable back to them. Data collection was conducted in three phases between October and December 2025, with a two-week interval between each phase; participants were required to complete each phase before proceeding to the next. In the first phase, participants completed questionnaires on demographic information and AI usage. A total of 463 questionnaires were completed at T1. In the second phase, the same respondents were invited to complete questionnaires on job complexity and perceived work meaningfulness, and 429 questionnaires were completed, indicating an attrition of 34 participants from T1 to T2. In the third phase, participants completed the questionnaire on employee resilience, and 400 questionnaires were completed, indicating an attrition of 29 participants from T2 to T3. Responses across the three waves were matched using anonymous participant IDs generated by the Credamo platform. After matching and deleting invalid responses, including cases that failed the occupational screening criteria, could not be successfully matched across the three waves, had substantial missing data, failed attention-check items, were completed in an unrealistically short time, or showed patterned responses, 332 valid matched questionnaires were retained for final analysis, resulting in a valid matched response rate of 83.0%. Table 1 presents the demographic characteristics of the final sample.

**Table 1:** Demographic variables.

Demographic characteristic	NO.	%
Gender		
Male	92	27.71
Female	240	72.29
Age (years)		
Below 20	4	1.20
21–30	138	41.60
31–40	159	47.90
41–50	21	6.30
51 and above	10	3.00
Education level		
College diploma and below	21	6.30
Bachelor’s degree	227	68.40
Master’s degree	78	23.50
Doctoral degree	6	1.80
Years of working experience (years)		
Less than 5	112	33.70
6–10	149	44.90
11–15	45	13.60
16–20	6	1.80
More than 21	20	6.00

### 3.2 Measures

All variables, with the exception of control variables, were rated on a five-point Likert scale, ranging from 1 (strongly disagree) to 5 (strongly agree). All measurement items in this study were adopted from well-established, peer-reviewed scales, and the original literature sources for each construct are clearly cited in the subsequent subsections. To enhance respondents’ comprehension and reduce contextual misunderstanding, each scale was equipped with a concise contextual introduction tailored to the participants. For example, clear definitions and practical workplace examples of AI tools were provided in the AI usage section, and contextual explanations were supplemented for job complexity to fit the actual work scenarios of financial staff. Specifically, in this study, AI usage refers to the extent to which accounting and finance employees apply AI-enabled tools in core daily work, including automated financial reporting, fraud detection, algorithmic risk assessment, automatic account reconciliation, audit tracking, anomaly identification, and intelligent decision support. These AI applications support employees in completing both routine and highly cognitive professional tasks to improve work efficiency and accuracy, excluding general-purpose or non-job-related AI tools.

All original English items were translated into Chinese and revised via a standard back-translation procedure to ensure semantic accuracy and cross-cultural consistency (Brislin, 1970). Minor wording adjustments were made to all the items to make them suitable for the domestic organizational environment and professional characteristics of Chinese financial practitioners.

#### 3.2.1 AI usage

AI Usage was measured using a three-item scale developed by Zang et al. (2024), with a sample item: “I use artificial intelligence to perform most of my work tasks.” Cronbach’s *α* = 0.769.

#### 3.2.2 Perceived work meaningfulness

PWM was measured using four items developed by Arnold et al. (2007). A representative item is “The work I do in this job is fulfilling.” One item—"I do not achieve important outcomes from the work I do in this job”—was reverse-scored. Cronbach’s α = 0.742.

#### 3.2.3 Job complexity

JC was measured using three items adapted from Cammann et al. (1983) and referenced to Shaw and Gupta (2004). A sample item reads: “My job is very complex.” Cronbach’s *α* = 0.779.

#### 3.2.4 Employee resilience

ER was measured based on the scale developed by Näswall et al. (2015), with a sample item: “I effectively collaborate with others to handle unexpected challenges at work.” Cronbach’s *α* = 0.709.

## 4 Results

### 4.1 Common method bias (CMB)

Harman’s single-factor test was applied to check for CMB. The rotation yielded five factors with eigenvalues exceeding 1 when all variables were pooled together. The findings indicated that the factor with the highest eigenvalue accounted for 26.544% of the variance (below the 40% threshold), suggesting that common method variance was unlikely to be a dominant concern. Furthermore, we estimated a model including an additional latent factor to capture potential common method variance. In this model, each item was allowed to load on its corresponding theoretical construct, while the additional latent factor was allowed to load on all observed items to capture shared method variance. The model showed acceptable fit (CFI = 0.893, TLI = 0.852, RMSEA = 0.073, SRMR = 0.056). Compared with the four-factor model (CFI = 0.946, TLI = 0.935, RMSEA = 0.048, SRMR = 0.051), the absolute changes in model fit were |ΔCFI| = 0.053, |ΔTLI| = 0.083, |ΔRMSEA| = 0.025, and |ΔSRMR| = 0.005. These changes were within acceptable ranges, with |ΔCFI| and |ΔTLI| below 0.10 and |ΔRMSEA| and |ΔSRMR| below 0.05. These results indicate that common method variance was unlikely to substantially bias the observed relationships.

### 4.2 Confirmatory factor analysis

The hypothesized model of this study specified four factors (AI usage, JC, PWM, and ER). Subsequently, three rival models were constructed, including a three-factor model (AI usage; JC + PWM; ER), a two-factor model (AI usage + JC + PWM; ER), and a single-factor model (AI usage + JC + PWM + ER). Table 2 shows the model fit indices. Among all tested models, the four-factor model had the best fit. This result supports discriminant validity across the study variables.

**Table 2:** Model fit.

Model	*χ* ^2^	df	*χ*^2^/df	RMSEA	SRMR	IFI	TLI	CFI
Four-factor model	249.778	141	1.771	0.048	0.051	0.947	0.935	0.946
Three-factor model	687.934	146	4.712	0.106	0.0876	0.736	0.687	0.733
Two-factor model	839.717	148	5.674	0.119	0.094	0.663	0.606	0.659
Single-factor model	948.466	149	6.366	0.127	0.097	0.611	0.548	0.606

In addition, composite reliability, convergent validity, and discriminant validity were assessed using CR, AVE, and HTMT. The CR values ranged from 0.766 to 0.838, exceeding the commonly recommended threshold of 0.70 (Hair et al., 2010) and indicating acceptable composite reliability. The AVE values ranged from 0.366 to 0.550. Although the AVE values for AI Usage, PWM, and ER were below the conventional threshold of 0.50 (Hair et al., 2010), their corresponding CR values exceeded 0.70. Following Fornell and Larcker (1981), constructs with AVE values below 0.50 may still be considered to have acceptable convergent validity when their composite reliability is adequate. Therefore, the convergent validity evidence was considered acceptable in light of the adequate CR values. To further assess discriminant validity, HTMT ratios were calculated. The HTMT values ranged from 0.194 to 0.813, all below the strict threshold of 0.85 recommended by Henseler et al. (2015), providing additional support for discriminant validity.

### 4.3 Descriptive statistics and correlations

Table 3 presents the descriptive statistics and correlations. AI usage was positively correlated with JC (*r* = 0.147, *p* < 0.01), PWM (*r* = 0.420, *p* < 0.01), and ER (*r* = 0.438, *p* < 0.01). JC was positively correlated with PWM (*r* = 0.187, *p* < 0.01) and ER (*r* = 0.274, *p* < 0.01). PWM and employee resilience demonstrated a strong positive correlation (*r* = 0.624, p < 0.01).

**Table 3:** Descriptive statistical analysis (*N* = 332).

Variable	*M*	SD	gender	age	edu	work	AI Usage	JC	PWM	ER
Gender	1.720	0.448	1.000							
Age	2.680	0.741	0.081	1.000						
Edu	2.210	0.573	−0.022	0.077	1.000					
Work	2.020	1.044	0.073	0.814**	−0.025	1.000				
AIU	3.622	0.799	−0.032	−0.009	0.175**	−0.060	1.000			
JC	3.745	0.782	−0.021	0.071	0.078	0.091	0.147**	1.000		
PWM	4.268	0.466	−0.095	0.104	0.091	0.096	0.420**	0.187**	1.000	
ER	4.30	0.354	−0.034	0.151**	0.150**	0.124*	0.438**	0.274**	0.624**	1.000

***p < 0.001, **p < 0.01, *p < 0.05; AI Usage, AIU; PWM, Perceived work meaningfulness; JC, Job Complexity; ER, Employee Resilience.

### 4.4 Testing of hypotheses

Before testing the hypotheses, multicollinearity diagnostics were conducted for the full regression models. Although age and work experience were strongly correlated (*r* = 0.814, *p* < 0.01), the VIF values ranged from 1.019 to 3.077, and the tolerance values ranged from 0.325 to 0.982. These values were within commonly accepted thresholds, suggesting that multicollinearity did not pose a serious threat to the regression estimates. Therefore, both age and work experience were retained as control variables because they capture conceptually distinct characteristics: chronological age and accumulated job-related experience.

Hypotheses were first tested using hierarchical regression, and Table 4 reports unstandardized coefficients, with standard errors shown in parentheses. Model 2 showed that AI usage positively predicted PWM [*b* = 0.246, SE = 0.029, 95% CI (0.188, 0.303), *p* < 0.001], supporting H2. AI usage positively predicted ER [*b* = 0.191, SE = 0.022, 95% CI (0.148, 0.234), p < 0.001 in Model 6], supporting H1. PWM also significantly predicted ER [*b* = 0.396, SE = 0.035, 95% CI (0.327, 0.465), *p* < 0.001 in Model 7], supporting H3 and suggesting that PWM may serve as a mediating mechanism. Table 5 shows that AI usage had a total effect of 0.179 [95% CI (0.135, 0.223)] on ER. The indirect effect was 0.090 with a 95% confidence interval [0.057, 0.127], excluding zero. Additionally, the indirect effect represented 50.3% of the total effect (0.090/0.179), indicating that PWM accounted for half of the impact of AI usage on ER, which supports H3.

**Table 4:** Results of hierarchical linear regression.

Variable	PWM				ER		
	Model 1	Model 2	Model 3	Model 4	Model 5	Model 6	Model 7
Gender	−0.106 (0.057)	−0.095 (0.052)	−0.092 (0.051)	−0.075 (0.051)	−0.034 (0.043)	−0.025 (0.039)	0.012 (0.033)
Age	0.037 (0.060)	0.019 (0.055)	0.022 (0.054)	0.025 (0.053)	0.053 (0.045)	0.038 (0.041)	0.031 (0.035)
Edu	0.069 (0.045)	0.013 (0.041)	0.007 (0.041)	−0.021 (0.042)	0.088* (0.034)	0.044 (0.031)	0.039 (0.026)
Work	0.025 (0.042)	0.046 (0.039)	0.039 (0.038)	0.034 (0.038)	0.014 (0.032)	0.030 (0.029)	0.011 (0.025)
AIU		0.246*** (0.029)	0.236*** (0.030)	0.235*** (0.029)		0.191*** (0.022)	0.094*** (0.021)
PWM							0.396*** (0.035)
JC			0.068* (0.030)	0.080** (0.030)			
AIU × JC				−0.124** (0.037)			
*R* ^2^	0.029	0.200	0.213	0.239	0.045	0.223	0.440
Δ*R*^2^	0.029	0.171	0.013	0.026	0.045	0.179	0.217
*F*	2.450*	16.295***	14.625***	14.517***	3.811**	18.740***	42.625***
Δ*F*	2.450*	69.619***	5.198*	11.156**	3.811**	75.004***	126.095***

Unstandardized coefficients are reported; standard errors are shown in parentheses. ***p < 0.001, **p < 0.01, *p < 0.05. AIU, AI usage; PWM, perceived work meaningfulness; JC, job complexity; ER, employee resilience.

**Table 5:** Breakdown of total, direct, and indirect effects.

Paths: AI usage → perceived work meaningfulness → employee resilience				
Effect type	Effect value	Standard error	95% confidence interval	
Total effect	0.179	0.023	0.135	0.223
Direct effect	0.089	0.021	0.048	0.130
Indirect effect	0.090	0.018	0.057	0.127

**Figure 2:**
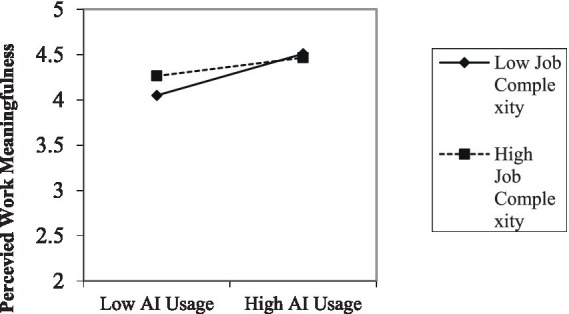
The moderating effect of job complexity.

H4 predicted a moderating effect of JC. The moderating effect was examined using Hayes’ PROCESS macro Model 1 in SPSS. Prior to estimating the interaction term, AI usage and JC were mean-centered, and the results are reported using unstandardized coefficients. As shown in Model 4, the interaction term between AI usage and JC negatively predicted PWM (*b* = −0.124, SE = 0.037, *p* = 0.001), supporting H4. To further interpret this interaction, simple slope analysis was conducted using PROCESS. The conditional associations between AI usage and PWM were estimated at low and high levels of JC (±1 SD from the mean). Figure 2 illustrates the moderating effect.

H5 proposes that JC moderates the indirect association between AI usage and ER via PWM, with the indirect association being stronger when JC is low. The moderated mediation model was tested using Hayes’ PROCESS macro Model 7 in SPSS. Bootstrap confidence intervals were based on 5,000 resamples. As shown in Table 6, at the low level of JC (−1 SD), the estimated indirect association between AI usage and ER via PWM was 0.132 [95% bootstrap CI (0.086, 0.189)]; at the high level of JC (+1 SD), this indirect association decreased to 0.055 [95% bootstrap CI (0.020, 0.094)]. More importantly, the index of moderated mediation was significant [Index = −0.049, BootSE = 0.018, 95% bootstrap CI (−0.088, −0.018)]. Because the bootstrap confidence interval did not include zero, the conditional indirect associations differed significantly across levels of JC, supporting H5.

**Table 6:** Moderated mediating effect test results.

Job complexity	Effect	BootSE	BootLLCI	BootULCI
Low JC (−1 SD)	0.132	0.026	0.086	0.189
Mean JC	0.093	0.018	0.061	0.131
High JC (+1 SD)	0.055	0.019	0.020	0.094
Index of moderated mediation	−0.049	0.018	−0.088	−0.018

## 5 Discussion

Drawing on JD-R theory, this study examined the relationship between AI usage and ER among accounting and finance employees and considered the boundary condition of JC. Based on 332 valid responses, three main findings emerged.

First, AI usage positively predicted both PWM and ER. Employees who used AI tools more frequently in their accounting and finance work tended to report a stronger sense of work meaningfulness and higher resilience when facing work pressure and uncertainty. This finding suggests that AI usage may operate as a work-related resource in digitally transforming accounting and finance contexts.

Second, PWM served as a mediator in the relationship between AI usage and ER. More frequent AI usage was linked to stronger perceptions that one’s work was purposeful, valuable, and meaningful, and these perceptions were further related to higher ER. This result points to PWM as an important psychological process connecting AI-supported work with employees’ resilience-related experiences.

Third, JC moderated the relationship between AI usage and PWM. The positive relationship between AI usage and PWM was stronger when JC was low and became weaker as JC increased. This finding suggests that the psychological significance of AI usage may depend on the nature of the job itself. In lower-complexity accounting and finance roles, AI may make work feel more meaningful by reducing repetitive tasks; in higher-complexity roles, employees may already derive meaning from professional judgment, autonomy, and complex problem-solving.

### 5.1 Theoretical contributions

First of all, this study addresses a research gap concerning the psychological mechanisms associated with AI usage. Previous studies have mainly examined AI in relation to efficiency and productivity (Zhang and Wei, 2025; Kassa and Worku, 2025), whereas less attention has been paid to employees’ psychological processes and work-related experiences. This study incorporates ER into the result variable and verifies the intermediary role of PWM, thus revealing the internal path correlation of the psychological adaptability of AI technology to employees (especially the group of accounting and financial practitioners who face the special pressure brought by algorithmic decision-making and automated reporting systems in daily work). Therefore, it is more realistic and targeted.

Secondly, existing studies have affirmed the positive role of AI technology in creating a supportive and creative working environment (Aljuhmani and Neiroukh, 2024). This study further shows that JC plays a key moderating role in the relationship between AI usage and PWM, thereby identifying the specific conditions under which AI usage is more strongly associated with employees’ psychological resilience and providing new empirical evidence on the boundary conditions of AI application.

The traditional JD-R framework is mainly used in the routine workplace (Bakker and Demerouti, 2007; Hobfoll, 1989). This study introduces the JD-R model into AI-driven work situations and extends the application boundaries of the model. Research shows that AI may function not only as a work resource that helps employees respond to workplace challenges, but also as a potential source of cognitive load and adaptation pressure. Its psychological implications may therefore depend on specific job demands and work contexts.

### 5.2 Managerial implications

First, organizations should actively incorporate AI adoption as a strategic resource for supporting ER among accounting and finance employees. This includes investing in intelligent auditing assistants, automated reporting systems, and AI-driven risk analysis platforms, alongside structured training programs and peer learning initiatives to build employees’ confidence and competency with these technologies.

Second, when implementing AI in work scenarios with different levels of complexity, organizations should tailor their AI application strategies to the specific nature of the tasks. For low-complexity tasks, such as routine bookkeeping and standardized financial reporting, AI can be used to reduce repetitive work and allow employees to focus on higher-value activities, including financial analysis and client consulting, thereby strengthening employees’ perceived work meaningfulness. In contrast, for complex roles such as tax planning and cross-border compliance, organizations should establish appropriate technical support and feedback mechanisms to ensure that AI assists rather than undermines employees’ professional judgment.

Beyond technical arrangements, employees’ psychological experiences during AI adoption should also be considered. From an organizational management perspective, companies should create a psychologically safe environment that encourages finance and accounting employees to adopt AI actively. At the same time, clear communication about the overall direction of AI strategy, especially regarding job restructuring and career development prospects, may help strengthen employees’ sense of professional purpose and reduce career anxiety during digital transformation.

### 5.3 Limitations and future research directions

It should be acknowledged that this study has several limitations. First, although a three-stage time-lagged design was adopted to reduce potential common method bias, this research design does not allow for definitive causal conclusions. While the survey design accounted for the temporal sequence of AI usage, PWM, JC, and ER, the directionality of the relationships among these variables still requires further validation. Future studies could employ longitudinal panel designs, experimental approaches, or intervention-based designs to more accurately capture how employees’ psychological responses to AI usage evolve over time.

Second, data were collected via self-reported employee questionnaires. Although this method is suitable for assessing subjective perceptions, respondents’ answers may still be influenced by social desirability bias, recall errors, or individual response tendencies. Future research could integrate survey data with objective indicators of AI usage, such as system logs, task records, or performance metrics. Qualitative research methods, including interviews, diary studies, or case analyses, could also provide deeper insights into how accounting and finance professionals experience AI-assisted work environments in their daily practices.

Third, this study primarily focused on JC as a boundary condition. Other contextual and individual factors may also influence the psychological implications of AI usage. Future research could explore moderating variables such as professional identity threat, digital self-efficacy, readiness for AI adoption, organizational support, or prior experience with intelligent technologies. A more in-depth analysis of these factors would help achieve a more comprehensive understanding of when and for which groups AI usage is more strongly associated with psychological benefits.

## 6 Conclusion

Drawing on JD-R theory, this study investigated how AI usage relates to ER among accounting and finance employees. Using data from 332 employees, the findings showed that AI usage was positively associated with ER and that PWM served as a mediating mechanism underlying this association. The indirect association was stronger among employees in low-complexity positions, suggesting that the psychological value of AI usage may vary across different job contexts. By extending the JD-R framework to AI-enabled accounting and finance work, this study contributes to a better understanding of how technology use is linked to employees’ work meaning and resilience.

## Data Availability

The raw data supporting the conclusions of this article will be made available by the authors, without undue reservation.
